# The *Phytophthora cactorum* genome provides insights into the adaptation to host defense compounds and fungicides

**DOI:** 10.1038/s41598-018-24939-2

**Published:** 2018-04-25

**Authors:** Min Yang, Shengchang Duan, Xinyue Mei, Huichuan Huang, Wei Chen, Yixiang Liu, Cunwu Guo, Ting Yang, Wei Wei, Xili Liu, Xiahong He, Yang Dong, Shusheng Zhu

**Affiliations:** 1 State Key Laboratory for Conservation and Utilization of Bio-Resources in Yunnan, Yunnan Agricultural University, Kunming, 650201 China; 2 Key Laboratory for Agro-biodiversity and Pest Control of Ministry of Education, Yunnan Agricultural University, Kunming, 650201 China; 3 Nowbio Biotechnology Company, Kunming, 650201 China; 4 Yunnan Research Institute for Local Plateau Agriculture and Industry, Kunming, 650201 China; 5 Department of Plant Pathology, China Agricultural University, Beijing, 100083 China

## Abstract

*Phytophthora cactorum* is a homothallic oomycete pathogen, which has a wide host range and high capability to adapt to host defense compounds and fungicides. Here we report the 121.5 Mb genome assembly of the *P. cactorum* using the third-generation single-molecule real-time (SMRT) sequencing technology. It is the second largest genome sequenced so far in the *Phytophthora* genera, which contains 27,981 protein-coding genes. Comparison with other *Phytophthora* genomes showed that *P. cactorum* had a closer relationship with *P. parasitica*, *P. infestans* and *P. capsici*. *P. cactorum* has similar gene families in the secondary metabolism and pathogenicity-related effector proteins compared with other oomycete species, but specific gene families associated with detoxification enzymes and carbohydrate-active enzymes (CAZymes) underwent expansion in *P. cactorum*. *P. cactorum* had a higher utilization and detoxification ability against ginsenosides–a group of defense compounds from *Panax notoginseng*–compared with the narrow host pathogen *P. sojae*. The elevated expression levels of detoxification enzymes and hydrolase activity-associated genes after exposure to ginsenosides further supported that the high detoxification and utilization ability of *P. cactorum* play a crucial role in the rapid adaptability of the pathogen to host plant defense compounds and fungicides.

## Introduction

*Phytophthora cactorum* (Lebert & Cohn) J. Schröt is a devastating homothallic oomycete pathogen^1,2^, which produces both sporangia (asexual reproduction) and oospores (sexual reproduction) in its life cycle in the field^1^. Oospores can remain dormant in soil for many years, making them difficult to manage^1^. In addition, sexual reproduction may allow this pathogen to maintain high adaptability to its host and environment. *P. cactorum* has a broad range of hosts, which includes over 200 species of trees, ornamentals, and fruit crops^3^. Other *Phytophthora* species, such as *P. sojae* and *P. ramorum*, have a narrow range of hosts^1,4,5^. Wide host range of pathogens or insects may be partly due to a high detoxification ability against the defense compounds from their hosts^6,7^. *P. cactorum* is an important pathogen of *Panax* species. It causes leaf blight, stem canker, and root rot in these plants^1,8,9^. *Panax* species can synthesize a high level of ginsenosides, a group of defense compounds that account for more than 6% of dry biomass in the plants^10^. Previous studies found that ginsenosides could inhibit the growth of leaf pathogen of *Alternaria panax* and nonpathogenic *Trichoderma* spp, whereas the growth of root pathogens (*P. cactorum*, *Fusarium solani*, *Fusarium oxysporum*, and *Cylindrocarpon destructans*) could not be inhibited by ginsenosides at the physiological concentration in roots of *P. notoginseng* or *P. quinquefolius*^11,12^. Our experimental data demonstrated that wide host range species–*P. cactorum*, *P. capsici*, and *P. parasitica*–had higher utilization or detoxification ability against ginsenosides compared with narrow host pathogen *P. sojae* (Supplementary Figs 1 and 2).

It has been reported that detoxification pathways used by organisms against plant defense compounds are co-opted for pesticide tolerance^6^. Most oomycete fungicides, including dimethomorph, flumorph, pyraclostrobine, kresoxim-methyl, fluopicolide, cymoxanil, and metalaxyl-M, have been widely used for the control of *Phytophthora* infection. Our fungicide sensitivity test found that the wide host range pathogens showed a high tolerance ability against these fungicides compared with narrow host range species (Supplementary Fig. 3). Previous study also showed that *P. cactorum* had a stronger ability to obtain fungicide tolerance when being cultured in increasing concentrations of fungicide^13^. Proteomic analysis revealed that many proteins involved in the detoxification metabolic pathway are responsible for the tolerance of *P. cactorum* to fungicides^14^. Over the past decades, it is apparent that *P. cactorum* has gradually developed tolerance to many fungicides in the field^9,13,15–17^. The above described data implied that *P. cactorum* had a high ability to detoxify plant defense compounds or fungicides. Thus, *P. cactorum* can provide a good system to understand the genetic and molecular bases of how *Phytophthora* species adapt to the defense compounds of their hosts and the fungicides in the environment.

The genome of *P. cactorum* is highly heterozygous, and it is difficult to *de novo* assembly using the next-generation sequencing technology. Here we report the 121.5 Mb genome assembly of the *P. cactorum* using the third-generation single-molecule real-time (SMRT) sequencing technology to generate super long reads to facilitate the genome assembly process. The genome of *P. cactorum* is the second largest genome sequenced in the *Phytophthora* genus so far. Comparative analyses of *Phytophthora* genomes showed extensive expansion of genes encoding detoxification enzymes and carbohydrate-active enzymes (CAZymes). These data provide important references to investigate the adaptation process in *P. cactorum* to plant defense compounds and fungicides.

## Results and Discussion

### Genome sequencing, assembly and characterization of *Phytophthora cactorum* genome

Based on the 5.2 Gb PacBio single-molecule sequencing data, the 121.5 Mb reference genome was assembled using the PBcR pipeline^18^. This process resulted in 5,449 scaffolds with an N50 of 30.67 Kb. The lengths of 97.3% scaffolds were greater than 5 Kb (Table 1). The genome of *P. cactorum* is the second largest among the sequenced *Phytophthora* species, only smaller than *P. infestans* (~240 Mb)^19^, but larger than *P. lateralis* (~44 Mb)^20^, *P. capsici* (~64 Mb)^21^, *P. ramorum* (~65 Mb)^22^, *P. fragariae* (~73.68 Mb)^23^, *P. parasitica* (~64.5 Mb)^24^, and *P. sojae* (~95 Mb)^22^. Based on the protists dataset in BUSCO^25^, the genome captured 170 (79.1%) complete BUSCOs (Benchmarking Universal Single-Copy Orthologs). There were 36 (16.7%) missing BUSCOs (Table 1; Supplementary Table 1). The *P. cactorum* genome showed a highly syntenic relationship with the genomes of *P. infestans, P. sojae*, and *P. capsici* (Supplementary Fig. 4).

**Table 1:** Summary of genome assembly and annotation for the *P. cactorum* genome.

Assembly	
Assembled genome size (bp)	121,526,021
Genome-sequencing depth (×)	42.8
No. of contigs	5,449
N50 of contigs (bp)	30,670
Longest contig (bp)	1,025,155
GC content of the genome (%)	52.15
Completeness evaluation	
CEGMA	95.16%
BUSCO	79.1%*
Annotation	
Percentage of repeat sequences (%)	46.69
Repeat sequence length (bp)	56,743,788
No. of predicted protein-coding genes	27,981
Percentage of average gene length (bp)	1,692.53
Average exon length (bp)	363.33
Average exon per gene	3.45
Total intron length (bp)	12,218,887
tRNAs	6731
rRNAs	376
snRNAs	376
miRNAs	2
Family number	11,674
Genes in families	19,783

^*^Based on protists_ensembl database.

The combination of *de novo* prediction and homology-based comparisons resulted in the identification of 56.7 Mb repetitive elements in the *P. cactorum* genome (Table 1; Supplementary Table 2), accounting for about 46.7% of the assembled genomes. 45.3% of the repeats in the *P. cactorum* genome were transposable elements (TEs), of which 20.3% were long terminal repeats (LTR) (Supplementary Table 3). The *P. cactorum* draft genome has more repeat sequences than *P. capsici* (19%), *P. sojae* (39%), and *P. ramorum* (28%), but less than *P. infestans* (74%).

We predicted 27,981 protein-coding genes in the assembled genome following a combination of homology and *ab initio* methods (Table 1). The average coding length was 1692.53 bp, and the average exon per gene was 3.45. *P. cactorum* had a noticeable expansion of gene content compared to *P. capsici* (19,805), *P. infestans* (17,797)*, P. sojae* (16,988), and *P. ramorum* (14,451). The gene density in *P. cactorum* (241/Mb) was less than *P. capsici* (268/Mb), but was higher than other *Phytophthora* species (74/Mb in *P. infestans*, 179/Mb in *P. sojae*, and 222/Mb in *P. ramorum*). Gene structure-based evaluation was performed to confirm the annotation of protein-coding genes (Supplementary Figs S5 and 6b,c). The analysis of local gene density in *P. cactorum* showed that most genes with intergenic regions were 400 bp to 15 kb long. The main distribution of flanking distances is wider in *P. cactorum* but not the other three sequenced genomes (Supplementary Fig. 6a). In addition, 63% of the predicted genes (17,566) showed expression levels (FPKM > 0.05) with the alignment of ~2.2 Gb RNA-seq data^26^ to the our genome (Supplementary Table 4). In total, 25,225, 11,533, 10,480 and 13,287 of the predicted genes were assigned with a functional annotation in the NR, Swiss-Prot, KEGG, and InterProScan databases, respectively (Supplementary Table 5).

An overview of annotated ncRNA is shown in Supplementary Table 6. A total of 6,731, 5,947, 143, and 218 tRNAs were identified in *P. cactorum, P. infestans, P. sojae*, and *P. ramorum*, respectively. The numbers of Leu-tRNA, Glu-tRNA, and Pro-tRNA in *P. cactorum* were most abundant. 376 rRNAs and 376 snRNAs were predicted in *P. cactorum*. Two mature miRNAs and four potential target genes of these miRNAs were identified (Table 1; Supplementary Table 7). These four target genes encoded a nuclear pore complex protein, a poly(A) polymerase, an acid/auxin permease, and an unknown protein, respectively.

### Comparative genomics and evolution of *Phytophthora* species

**Figure 1:**
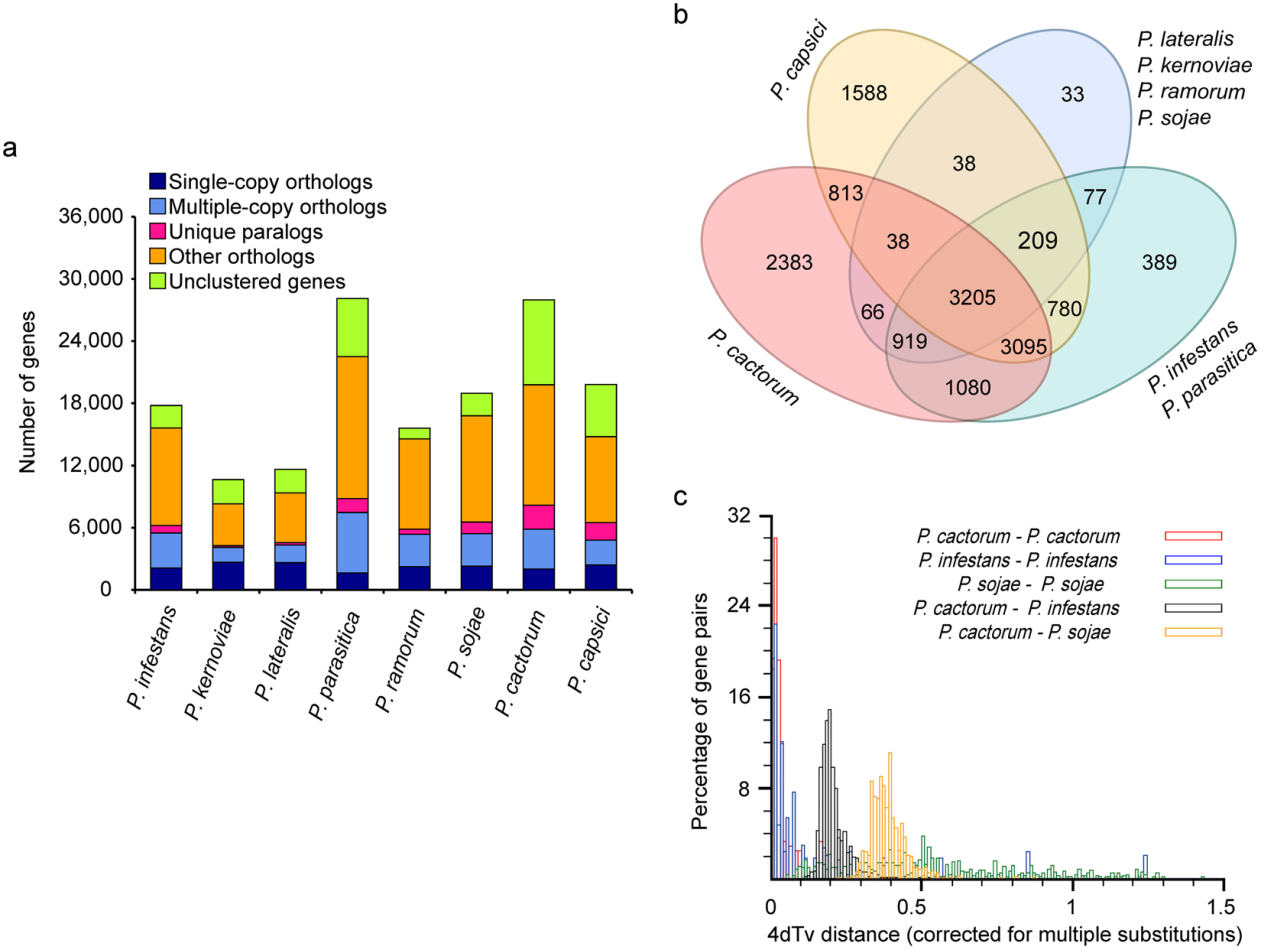
Evolutionary analysis of the *P. cactorum* genome assembly. (**a**) The orthologous gene distribution in eight sequenced *Phytophthora* species. (**b**) Venn diagram showing the number of unique and shared gene families among eight *Phytophthora* species. (**c**) Whole-genome duplications in *P. cactorum*, *P. infestans* and *P. sojae* as revealed by the distribution of 4DTv distance (four-fold degenerate third-codon transversion) between orthologous genes.

Gene family clustering analysis of eight *Phytophthora* species identified 11,674 gene families with a total of 19,783 genes in *P. cactorum* (Table 1; Fig. 1a). The numbers of single-copy orthologs in eight *Phytophthora* species were comparable. *P. cactorum* had 8,198 unclustered genes and 893 unique gene families (2,310 unique paralogs) (Supplementary Table 8). Among the genes unique to *P. cactorum*, the majority were enriched in defense response, cell cycle, interaction between organisms, peptidyl-amino acid modification, regulation of cell cycle, and TOR signaling pathway in the biological process (Supplementary Table 9). The Venn diagram showed that the eight *Phytophthora* species shared a common core set of 3,205 gene families (Fig. 1b). The number of *P. cactorum*-specific gene families was 2,383 (Fig. 1b).

**Figure 2:**
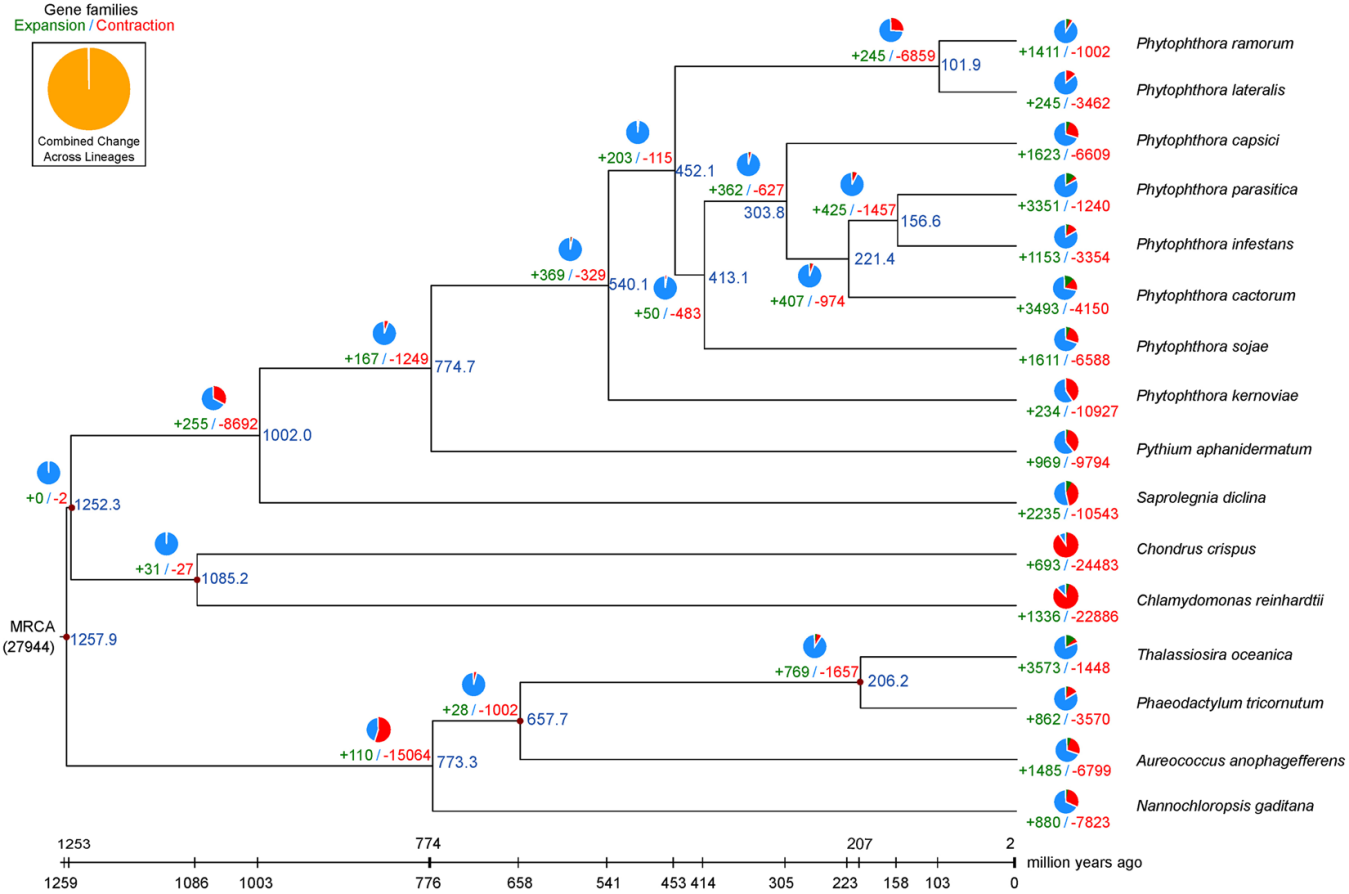
Phylogenetic relationship, the divergence time and gene contract and expand among oomycetes (*Phytophthora* sp, *Pythium aphanidermatum* and *Saprolegnia* diclina), stramenochromes [diatoms (*Thalassiosira oceanica, Phaeodactylum tricornutu*), *Aureococcus* (*Pelagomonadales*) and *Nannochloropsis* (*Eustigmatales*)], red algae (*Chondrus crispus*) and green algae (*Chlamydomonas reinhardtii*). The estimated divergence time was 221.4 (138.6–342.4) million years ago (MYA) between *P. parasitica*, *P. infestans* and *P. cactorum*. The number of contracting and expanding gene families among 16 species is shown in the pie chart, the estimates of divergence time are indicated at each node. The red dot on branches means divergence time has been adjusted by fossil evidence.

To systematically study the evolutionary dynamics of Stramenopile species, species phylogeny was performed utilizing 5,103 single-copy orthologous genes among 16 species, which included red algae (*Chondrus crispus*) and green algae (*Chlamydomonas reinhardtii*) (Fig. 2). The phylogenetic analysis revealed that red algae and green algae were grouped into one branch. The pathogenic oomycetes (such as *Phytophthora*, *Pythium*, and *Saprolegnia*) were separated from the nonpathogenic stramenochromes (such as *Thalassiosira*, *Aureococcus*, and *Nannochloropsis*). *P. cactorum* was more closely related to wide host range species (*P. parasitica*, *P. infestans*, and *P. capsici*) than to other sequenced *Phytophthora* species. Furthermore, the phylogenetic analysis mostly resembles the known topology of the tree of Stramenopile^27,28^. The exact topology of the eight *Phytophthora* species based on genomic data is highly consistent with the phylogenetic relationships of *Phytophthroa* species studies by Blair *et al*.^29^ and Runge *et al*.^30^ using multi-locus analysis. With the sequencing of more oomycete species genomes, the true phylogeny between Pythiaceae and Peronosporaceae in Peronosporales will gradually become clear. The estimated divergence time between *P. parasitica, P. infestans* and *P. cactorum* was 221.4 (138.6–342.4) million years ago (MYA) (Fig. 2). This most comprehensive and robust study of *Phytophthora* relationships to date based on genomic data will provide a phylogenetic framework for interpreting the evolutionary events of the genus.

The expansion of gene families was frequently reported to directly or indirectly involve in pathogenicity in fungal pathogens^19,22,28,31–33^. 3,493 and 4,150 gene families of *P. cactorum* had undergone expansion and contraction, respectively (Fig. 2). The number of expanded gene families in *P. cactorum* was the largest among *Phytophthora* species, whereas the number of contracted gene families was relatively small. The expanded genes in *P. cactorum* were enriched in membrane, cytoskeleton, transport, carbohydrate metabolism, nucleotide binding, transporter activity, transferase activity, and hydrolase activity (p < 0.01, Supplementary Table 10). The contracted genes in *P. cactorum* were enriched in cellular catabolism, DNA metabolism, chromosome, nucleic acid binding, and nucleotide binding (p < 0.01, Supplementary Table 11). The expansion of gene families is likely the result of the transposons^33^. The expansion of gene families in *P. cactorum* may be due to the large number of transposable elements in genome. However, more analysis should be performed to elucidate the relationship of transposons and gene family expansion.

Whole-genome duplication (WGD) followed by gene loss has been found in most eudicots and is regarded as the major evolutionary force that gives rise to gene neofunctionalization in both plants and animals^34^. It has been speculated that the increased genome size and gene content of *Phytophthora* species may be due to WGD or their divergent repertoires of transposable elements^19,35,36^. WGD analysis of three *Phytophthora* species indicated that both *P. cactorum* and *P. infestans* have experienced a recent WGD event (Fig. 1c). This WGD event helps clarify some of the evolutionary history of *Phytophthora* species. The time of divergence between *P. cactorum* and *P. infestans* was later than that between *P. cactorum* and *P. sojae*, which was consistent with the phylogenetic tree (Fig. 2). And some evidence indicated the WGD in *P. cactorum* was followed by gene loss (Supplementary Fig. 7).

Positive selection was proposed to contribute to fitness. About 428 to 634 *P. cactorum* genes were determined as positive-selected genes comparing with other *Phytophthora* species (p < 0.05; Supplementary Table 12 sheet 1–7). GO enrichments analysis demonstrated that most of these genes in *P. cactorum* were involved in cellular macromolecule metabolism, hydrolase activity, and transferase activity (Supplementary Table 13).

*P. cactorum, P. parasitica*, and *P. capsici* have a wide spectrum of hosts, and *P. sojae* has a narrow spectrum of hosts. It has been reported that oomycete pathogens have a large and diverse repertoire of expanded gene families^19,22,28,37^. Most of the expanded gene families were reported to be directly or indirectly involved in pathogenicity, such as glycoside hydrolases^28,38^ or secreted proteins characterized by the presence of either the RXLR or the LXLFLAK (Crinkler) motifs^19,39–41^. Comparative genomic analysis demonstrated that the numbers of pathogenicity-associated genes, including proteinase inhibitor, protein toxins, secretomes, secondary metabolites biosynthesis, were similar to or smaller than the numbers reported for *P. capsici*, *P. sojae*, and *P. parasitica*. The numbers of genes involved in CAZymes and detoxification metabolism were increased in the genome of *P. cactorum* (Table 2). Thus, the divergence of wide host range species and narrow host range species may be partly associated with the expansion and extraction of genes involved in detoxification enzymes and CAZymes.

**Table 2:** Potential infection-related genes in the *P. cactorum, P. sojae, P. capsici* and *P. parasitica*.

Gene product	*P. cactorum*	P. capsici*	P. parasitica*	*P. sojae*
**Proteases, all**	**87**	**40**	**64**	**186**
Serine proteases	47	18	40	119
Cysteine proteases	40	22	24	67
**Carbohydrate-active enzymes (CAZys)**	**901**	**628**	**839**	**786**
Glycosyl hydrolases (GHs)	374	261	312	125 (314)
Glycosyl transferases (GTs)	190	130	220	(155)
Polysaccharide lyases (PLs)	73	54	44	(58)
Auxiliary Activities (AAs)	50	43	50	48
Carbohydrate-binding modules (CBMs)	103	54	102	92
Carbohydrate esterases (CEs)	111	86	111	119
**Pectinases**	**68**	**55**	**54**	**62**
Pectin esterases	24	7	16	19
Pectate lyases	44	48	38	43
**Cutinases**	**7**	**6**	**6**	**16**
**Chitinases**	**3**	**2**	**4**	**5**
**Lipases**	**10**	**12**	**15**	**171**
**Phospholipases**	**55**	**29**	**44**	**>50**
**Protease inhibitors**	**17**	**25**	**30**	**19**
Kazal	14	23	28	15
Cystatin	3	2	2	4
**Protein toxins**	**41**	**45**	**51**	**48**
NPP family	37	39	49	29
PcF family	4	6	2	19
**Secondary metabolite biosynthesis**	**4**	**3**	**13**	**4**
Nonribosomal peptide synthetases	3	2	9	4
Polyketide synthases	1	1	4	0
**Effectors**	**174**	**156**	**294**	**218**
Elicitins	39	48	54	57
Avh (RXLR) family	135	108	240	350 (120)
Crn family (Crinklers)	16	25	13	40 (41)
**Detoxification metabolism**	**896**	**695**	**794**	**585**
ABC transporters (ABC)	60	40	48	134 (42)
Major facilitator superfamily (MFS)	239	217	242	228
Cytochrome P450’s (CYPs)	46	36	40	30 (33)
Alcohol dehydrogenase (ADH)	101	58	71	52
Short-chain dehydrogenase/reductase (SDR)	84	68	79	67
Peroxidase (POD)	56	31	35	34
Glutathione S-transferases (GSTs)	45	32	37	41
Methyltransferase (MTR)	265	213	242	163

* and () indicated the data obtained according our method.

For successful infection, phytopathogenic microorganisms have the ability to adapt to the plant defense system through detoxification or direct utilization of plant defense compounds^42,43^. In this study, we found that *P. cactorum* made good use of ginsenosides as the sole carbon source to growth (Supplementary Fig. 1). Four tested *Phytophthora* species showed similar abilities to utilize glucose, xylan, pectin, cellulose, and gum guar. However, *P. cactorum*, *P. capsici*, and *P. parasitica* showed higher ability to utilize ginsenosides than *P. sojae* (Supplementary Fig. 2). Ginsenosides can be hydrolyzed by microbial CAZY enzymes^44^. Based on genomic analysis, we predicted 901 genes that putatively encode CAZY enzymes in *P. cactorum*. This number was larger than those of other three sequenced *Phytophthora* species (Table 2). Especially, the members of GHs, GT, and PL families were expanded in *P. cactorum*.

Microbes have evolved the ability to detoxify xenobiotics through enzymes and transporters^45–47^. We identified the ATP-binding cassette (ABC) transporter families and major facilitator superfamily (MFS), as well as the cytochrome P450 (CYPs), peroxidase (POD), glutathione S-transferases (GSTs), methyltransferase (MTR), and dehydrogenase in the *P. cactorum* genome (Table 2). Our previous proteomic analysis also showed that *P. cactorum* employed detoxification enzymes to tolerate a series of fungicides^13^. These data imply that the expansion of detoxification metabolism genes enable *P. cactorum* with higher detoxification ability against host defense compounds or commercial fungicides. However, more genetic studies should be performed to elucidate the function and relationship of these key genes.

### Transcriptome

**Figure 3:**
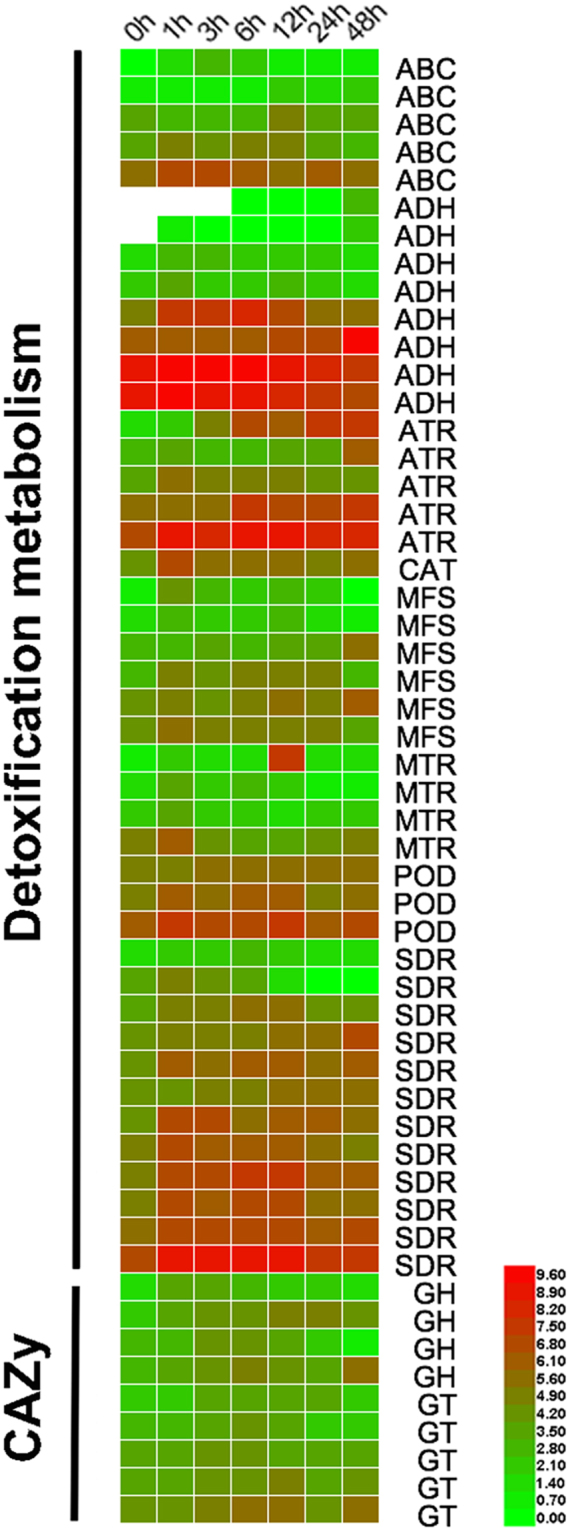
Heat-map depicting the changes of the up-regulated expressed genes involved in detoxification metabolism and CAZymes after exposure to ginsenosides in time-course treatments of *P. cactorum*. ABC, ABC transporter; MFS, major facilitator superfamily; ADH, alcohol dehydrogenase; SDR, short-chain dehydrogenase/reductase; MTR, methyltransferase; ATR, acyltransferase; CAT, catalases; POD, peroxidase; GH, glycoside hydrolase; GT, glycosly transferase. Detailed descriptions of these metabolites are shown in Supporting Information Table S18.

Transcriptomes from four *Phytophthora* species (*P. cactorum*, *P. capsici*, *P. parasitica*, and *P. sojae*) exposed to ginsenosides for 24 hours contained a total of 267 (Supplementary Table 14), 408 (Supplementary Table 15), 18 (Supplementary Table 16), and 28 (Supplementary Table 17) differentially expressed genes (DEGs), respectively (Supplementary Fig. 8). A set of the up-regulated DEGs in *P. cactorum* and *P. capsici* were involved in the detoxification metabolism and glycosyl hydrolase, highlighting the potential roles of these genes in adaptation to ginsenosides. Furthermore, time-course transcriptome analyses respectively identified 179, 355, 45, 270, 341, and 127 DEGs in *P. cactorum* after exposure to ginsenosides for 1 h, 3 h, 6 h, 12 h, 24 h, and 48 h (Supplementary Fig. 9). The GO classifications showed that nine GO terms were unique for up-regulated genes, which included drug binding and transporter, transcription related factors, and enzyme activator GO terms (Supplementary Fig. 10). Further analyses showed that a series of glycoside hydrolase and transferase genes as well as detoxification-related genes were induced after the exposure to ginsenosides (Fig. 3; Supplementary Tables 18 and 19). Previous study demonstrated that ginsenosides could be hydrolyzed by microbial glycosyl hydrolases to release glycosyl as nutrient for microbes^44^. Although the function of ginsenosides induced detoxification-related genes in *P. cactorum* should be proven by further genetic studies, these genes were frequently reported in chemoresistance^45–47^. Thus, *P. cactorum* could not only detoxify ginsenosides through detoxification-related enzymes but also utilize them as nutrient for growth through glycosyl hydrolases. It may be an important strategy for *P. cactorum* to infect *P. notoginseng*.

Transcription factors regulate gene expression and protein kinases regulate cellular activities by phosphorylating target proteins in response to internal or external signals. We identified a total of 566 transcription factors and 536 protein kinases in the *P. cactorum*. The numbers were smaller than those found in *P. parasitica* (689 transcription factors, 577 protein kinases), but larger than those found in *P. capsici* (458, 406), *P. sojae* (431, 423), *P. infestans* (381, 413), *P. ramorum* (367, 399), and *P. kernoviae* (252, 231) (Supplementary Tables 20 and 21). The C2H2, MYB-related and SET transcription factors were comparatively abundant in *P. cactorum*, as well as the group CAMK and TKL protein kinases. *P. cactorum* has an expanded RLK Pelle group proteins of 22 members. There were also a large number of unclassified kinases, suggesting novel functions performed by the *P. cactorum*. After exposure to ginsenosides, a set of transcription factors and protein kinases-related genes were significantly up- or down- regulated (Supplementary Tables 18 and 19), which may facilitate the adaptation of *P. cactorum* to defense compounds.

## Conclusions

In summary, we sequenced the *P. cactorum* using the third-generation single-molecule real-time (SMRT) sequencing technology and revealed the relationship between *P. cactorum* and other sequenced *Phytophthora* species. Comparative genomics analyses identified the expansion of gene families associated with the detoxification and carbohydrate-active enzymes (CAZymes) against plant defense compounds. These genes may enable *P. cactorum* with a high ability to tolerate or utilize plant defense compounds and commercial fungicides. This may partly explain the pathogenicity of *P. cactorum* in a broad range of hosts. Together, our genomic analyses provide insights into the adaptive mechanisms of *P. cactorum* to plant defense compounds and fungicides, which will facilitate future studies on pathogenesis and disease management.

## Methods

### DNA isolation, sequencing and assembly

*P. cactorum* was isolated from an infected *P. notoginseng* in Yunnan. The mycelia were harvested after the strains grown in 150 mL of carrot liquid medium in a 500-mL shaker culture flask on a shaker for three days at room temperature, respectively. Then genomic DNA used for sequencing was extracted from mycelia using Omega Fungal DNA Kit according to the manufacturer’s instructions. Briefly, fresh fungal tissue was disrupted and then lysed in a specially formulated buffer containing detergent. Contaminants were further removed after DNA precipitation using isopropanol. Binding conditions were then adjusted and the sample was applied to a spin-colum. Trace contaminants such as residual polysaccharides were removed with two rapid wash steps, and pure DNA was eluted using low ionic strength buffer. In total of 50 mg DNA were used to construct the PacBio sequencing libraries.

Then sequencing was performed to produce raw reads. Totally, 4.84 Gb filtered subreads were obtained for *P. cactorum* from raw data. Though the error rates of single-molecule reads are high, but we yielded a considerable amount of long reads (43×), which required for self-correction and for subsequent *de novo* assembly, to assemble the genome. Automatic assembly was performed using PBcR pipeline of Celera Assembler version 8.3rc1^18^. Syntenies was yielded through aligning the scaffolds of *P. cactorum* to that genome of *P. infestans, P. sojae* and *P. capsici* using NUCmer in MUMmer 3.23^48^, respectively.

### Repeats annotation

First, we searched for tandem repeats across the genome using the program Tandem Repeat Finder (TRF)^49^. The transposable elements (TEs) in the genome were identified by a combination of homology-based and *de novo* approaches. For homolog based prediction, known repeats were identified using RepeatMasker^50^ and RepeatProteinMask^50^ against Repbase^51^ (Repbase Release 16.10; http://www.girinst.org/repbase/index.html). Repeat Masker was applied for DNA-level identification using a custom library. At the protein level, RepeatProteinMask was used to perform an RMBLAST search against the TE protein database. For *de novo* prediction, RepeatModeler (http://repeatmasker.org/) and LTR FINDER^52^ were used to identify *de novo* evolved repeats inferred from the assembled genome.

### Gene prediction and functional annotation

We used the MAKER2^53^ pipeline to predict protein-coding genes in the genome. First, the genome was repeat masked using the result of repeat annotation. Then the masked genome was fed to the MAKER2 pipeline with the *ab initio* gene predictors being GeneMark-ES, FGENESH, Augustus and SNAP. And for the evidence-driven gene prediction, cDNA of *P. infestans* and proteins of six related species from Ensembl (release-28, *P. infestans*, *P. kernoviae*, *P. lateralis*, *P. parasitica*, *P. ramorum*, and *P. sojae*) were fed to the pipeline. Gene functions were assigned according to the best match alignment using BLASTp against NR, Swiss-Prot and KEGG databases. InterProScan functional analysis and Gene Ontology IDs were obtained using InterProScan^54^.

### Non-coding gene annotation

Software tRNAscan-SE^55^ is specified for Eukaryotic tRNA and was deployed for tRNA annotation. We used homologous method to identify rRNA. The rRNA sequence data downloaded from Rfam^56^ database was used as a reference. INFERNAL^57^ was used to identify snRNA.

### Gene family cluster

To identify different sets of gene clusters, protein-coding genes sequences of 16 species were downloaded from Esembl and JGI (http://genome.jgi.doe.gov) and used to locate gene clusters. After pairwise aligning was conducted using BLASTp with an E-value cutoff of 10^−5^, OrthoMCL package^58^ was performed to identify the gene family clusters using the BLASTp output with default parameters, final paralogous and orthologous genes were defined using MCL software in OrthoMCL.

### Phylogenetic tree construction

The single-copy orthologous genes defined by OrthoMCL^58^ were formed, then multiple single-copy genes was aligned using MUSCLE^59^, and the aligned sequences were extracted to feed to MrBayes (http://mrbayes.sourceforge.net) to inferred the species phylogeny using a maximum likelihood (ML) approach. To estimate the divergence time of each species, the information about the already known divergence time data between these species from http://www.timetree.org/ were collected. The topology of the ML tree was fed to MCMCTREE in paml version 4.4^60^ for constructing a divergence time tree and calculated the divergence time. Based on the calculated phylogeny and the divergence time, CAFÉ^61^ (Computational Analysis of Gene Family Evolution, version 2.1), a tool based on the stochastic birth and death model for the statistical analysis of the evolution of gene family size, was applied to identify gene families that had undergone expansion and/or contraction. The GO enrichment was done with Ontologizer 2.0^62^ by using one-sided Fisher’s exact test, the Parent-Child-Union method, with a p-value cut-off of 0.01. All genes with GO annotation were used as reference, and the genes undergone expansion or contraction was used as study set.

### Detection of positively selected genes

To detect genes under positive selection, BLASTn was performed to align the coding sequence (CDS) libraries of *P. infestans, P. lateralis, P. capsici, P. ramorum, P. kernoviae, P. parasitica* and *P. sojae* against the *P. cactorum* CDS library, respectively, in order to find the gene pairs with the best alignments. The resulting orthologous gene pairs were aligned again using lastz with the default parameters as a preparation for KaKs_Calculator 1.2^63^, which finally yielded a dataset of each gene pair’s Ka/Ks ratio, and the Ka/Ks ratio >1 was determined a positively selected gene (significance, p-value < 0.05). The GO enrichment was done with Ontologizer 2.0^62^ by using one-sided Fisher’s exact test, the Parent-Child-Union method, with a p-value cut-off of 0.05. All genes of *P. cactorum* were used as reference, and of all positively selected genes in *P. cactorum* were used as study set.

### Characterization of protein families

Transcription factors and protein kinases were identified using iTAK v1.5 (http://bioinfo.bti.cornell.edu/cgi-bin/itak/index.cgi). Carbohydrate-active enzymes (CAZymes) were identified by scanning using HMMER 3.0^64^ against the Hidden Markov Model (HMMs) corresponding to the Pfam^65^ CAZyme family and subfamily (download from http://csbl.bmb.uga.edu/dbCAN/)^66^. Secondary metabolism genes were annotated based on their genomic context and domain content using an automatic web-based software SMURF (www.jcvi.org/smurf/)^67^.

The gene families of potential infection-related genes were scanned using HMMER with HMMs against the Pfam families (E-value cutoff of 0.01, PF00067 for cytochrome P450, PF05630 for NPP1 family). The candidates of NPP1 family were further identified to confirm the existence of a signal peptide in N-terminal. Transporters were identified by scanning for the PFAM domains representing both two ABC transporters domains (PF00005 and PF00664) and assisted with manual inspection. Statistics of other proteins in Table 2 were based on the annotation of InterProScan database.

The families of CRN effectors in *P. cactorum, P. sojae, P. capsici* and *P. parasitica* dataset were initially predicted based on the BLASTp comparisons (E-value cut-off of 10^−5^) against the collection of CRN effectors of *Phytophthora* species and NCBI databases, and confirmed the existence of a signal peptide in N-terminal.

### RXLR effector prediction

A reference method^68^ to identify sequences containing a signal peptide and the predicted cleavage site must be within first 40 amino acids in N-terminal using SignalP4.0^69^, the RXLR motif was extended to incorporate the presence of an [ED][ED][KR] motif down-stream and within 40 amino acids of the RXLR motif. The RXLR position must be downstream of the signal peptide cleavage site, and the RXLR motif and [ED][ED][KR] motif must be within the first 100 amino acids downstream of the signal peptide cleavage site.

### The sensitivity test of *Phytophthora* species to plant defense compounds and fungicides

The cultures of *P. cactorum*, *P. capsici*, *P. parasitica*, and *P. sojae*, growing on carrot agar medium (CA) plates were transferred onto new CA plates amended with crude ginsensides at concentrations of 0, 0.10, 0.50, 1.0, 5.0, and 10.0 g L^−1^ or fungicides at the following concentrations: fluopicolide, dimethomorph and flumorph, at 0, 0.1, 0.5, 1.0, and 2.0 mg L^−1^; pyraclostrobine and kresoxim-methyl, at 0, 0.1, 0.5, 1.0, 2.0, and 5.0 mg L^−1^; cymoxanil, at 0, 10, 20, 40, and 80 mg L^−1^; metalaxyl-M, at 0, 0.1, 1.0, 10.0, and 50.0 mg L^−1^. Fungicide or ginsenosides was dissolved in methanol (OmniSolv, HPLC grade) to prepare stock solutions. To prepare agar plates supplemented with serial dilutions of fungicides or ginsenosides, the stock solutions were added into CA medium (200 g boiled carrot and 15 g agar in a total volume of 1 L of distilled water) when CA medium was cooled to 50 °C. The final concentration of methanol in any tested media was limited to 0.1% (vol/vol). The experiment was performed three times with four replicates and incubated for 4 days in the dark at 25 °C. The diameters of the colonies were measured perpendicularly. The ginsenosides was extracted from three-year-cultivated *P. notoginseng* roots with MeOH:H_2_O (80:20) and identified by HPLC-MS as described previously^70^.

### Growth profiles of *Phytophthora* species on single carbon source

Citrus pectin, glucose, xylan, cellulose, gum guar and crude ginsenosides (all from Sigma) were used as single carbon source separately in agar medium, to evaluate the growth of different *Phytophthora* species. Inocula of *P. cactorum* and other *Phytophthora* species (*P. capsici*, *P. parasitica*, and *P. sojae*) were placed on these media and incubated at 25 °C for four days. These tests were repeated three times, and the results were analysed to evaluate mycelium growth ability on different single carbon source.

### RNA-seq

*P. cactorum* and other *Phytophthora* species (*P. capsici*, *P. parasitica*, and *P. sojae*) were grown from mycelial inocula at 27 °C (150 mL of carrot liquid medium in a 500-mL shaker culture flask) shaking at 115 rpm. 10 fresh plug (5 mm in diameter) was taken from the growing edge of a CA culture and transferred into 150 mL of medium in each shock culture flask, and the mixture was incubated in an orbital shaker (ZHWY-111B, Shanghai ZHICHENG Analytical Instruments Manufacturing Co., Ltd.) at 150 rpm at 25 °C. After 24 h pre-incubation, ginsenosides stock solution was added to the medium to a final concentration of 1.0 mg L^−1^. For the control culture, only methanol was added. Mycelia were collected at 24 h after exposure to ginsenosides. For *P. cactorum*, mycelia were collected at 1, 3, 6, 12, 24, and 48 h after treatment with ginsenosides. And then, the mycelial mat was separated from the medium by filtration, quickly washed three times with deionized water, then immediately frozen in liquid nitrogen and lyophilized. Each treatment had two independent replicates.

3 μg of total RNA per sample was used as input material for the RNA sample preparation. Beads with oligo (dT) were used to isolate poly(A) mRNA from total RNA. RNA sequencing libraries were constructed from these mRNA using the TruSeq RNA Sample Preparation Kit (Illumina, San Diego, USA). Briefly, the Elution 2-Frag-Prime (94 °C for 8 minutes, 4 °C hold) was used to elute, fragment and prime the mRNA with Elute, Prime, Fragment Mix (Illumina). First strand cDNA synthesis was performed with First Strand Master Mix and SuperScript II mix (ratio: 1 µl SuperScript II/7 µl First Strand Master Mix) (Invitrogen). The second strand was synthesized with Second Strand Master Mix (Illumina) and Ampure XP beads (Illumina) were used to separate the double-stranded (ds) cDNA from the 2nd strand reaction mix. After end repair and the addition of a 3′-dA overhang, the cDNA was ligated to Illumina PE adapter oligo mix (Illumina), and size-selected for 400 ± 10% bp fragments by gel purification. After 15 cycles of PCR amplification, the paired-end libraries were sequenced using the paired-end sequencing module (150 bp at each end) of the Illumina HiSeq 4000 platform.

These raw reads were processed through Trimmomatic (Version 0.32)^71^ to remove reads containing adapter, reads containing poly-N and low quality reads from the raw data and yielded clean data for downstream analyses. The corresponding trimmed clean reads were aligned to the related reference genome (*P. parasitica* and *P. sojae* were downloaded from Ensembl, and the *P. capsici* genome was downloaded from JGI database) employing TopHat2^72^ software with default settings. Calculation of gene expression level and identification of differentially expressed genes (DEGs) between the time-course (for *P. cactorum*) or treatments (for *P. parasitica*, *P. sojae* and *P. capsici*) and control groups were conducted using Cufflinks v2.2.1^73^. Fragments per kilobase of exon per million fragments mapped (FPKM) were used to normalize RNA-seq fragment counts and estimate the relative abundance of each gene. The Cuffdiff package in Cufflinks was used to perform pairwise comparisons of the expressions of each gene between treatments and control in the four species and to report DEGs and transcripts. The DEGs were decided based on a p-value < 0.05 and at least a 2-fold change between the two FPKM values.

### Data availability

All raw genome sequence data have been deposited in the Short Read Archive (SRA) at NCBI under accession number SRR3386345 (PRJNA318145). Raw RNA-seq data have been deposited in the SRA under accession number SRP111895.

## Electronic supplementary material

Supplementary Figures and Tables

Supplementary Dataset Information

Supplementary Table S4

Supplementary Table S5

Supplementary Table S6

Supplementary Table S9

Supplementary Table S10

Supplementary Table S11

Supplementary Table S12

Supplementary Table S13

Supplementary Table S14

Supplementary Table S15

Supplementary Table S16

Supplementary Table S17

Supplementary Table S18

Supplementary Table S19

Supplementary Table S20

Supplementary Table S21
