# Author Correction: The *Phytophthora cactorum* genome provides insights into the adaptation to host defense compounds and fungicides

**DOI:** 10.1038/s41598-020-64605-0

**Published:** 2020-05-01

**Authors:** Min Yang, Shengchang Duan, Xinyue Mei, Huichuan Huang, Wei Chen, Yixiang Liu, Cunwu Guo, Ting Yang, Wei Wei, Xili Liu, Xiahong He, Yang Dong, Shusheng Zhu

**Affiliations:** 1 State Key Laboratory for Conservation and Utilization of Bio-Resources in Yunnan, Yunnan Agricultural University, Kunming, 650201 China; 2 Key Laboratory for Agro-biodiversity and Pest Control of Ministry of Education, Yunnan Agricultural University, Kunming, 650201 China; 3 Nowbio Biotechnology Company, Kunming, 650201 China; 4 Yunnan Research Institute for Local Plateau Agriculture and Industry, Kunming, 650201 China; 5 Department of Plant Pathology, China Agricultural University, Beijing, 100083 China

Correction to: *Scientific Reports* 10.1038/s41598-018-24939-2, published online 25 April 2018

The information in this Article is incomplete. Since the publication of this Article the Whole Genome Shotgun project has been deposited at DDBJ/ENA/GenBank under the accession number WXXK00000000; the assembled genome and annotations has also been deposited in the Narional GeneBank DataBase (Project ID: CNP0000756). Therefore, the Data Availability text,

“All raw genome sequence data have been deposited in the Short Read Archive (SRA) at NCBI under accession number SRR3386345 (PRJNA318145). Raw RNA-seq data have been deposited in the SRA under accession number SRP111895.”

should read:

“All raw genome sequence data have been deposited in the Short Read Archive (SRA) at NCBI under accession number SRR3386345 (PRJNA318145). Raw RNA-seq data have been deposited in the SRA under accession number SRP111895. This Whole Genome Shotgun project has been deposited at DDBJ/ENA/GenBank under the accession WXXK00000000. The version described in this paper is version WXXK01000000, the gene IDs have been updated according to Supplementary Data (file: Phyca.WXXK01.p2g.xlsx). The assembled genome and its annotation also were deposited in the China National GeneBank DataBase (Project ID: CNP0000756).”

