# Supplementary material for: The Phytophthora cactorum genome provides insights into the adaptation to host defense compounds and fungicides
Source: Sci Rep. 2018 Apr 25;8:6534. doi: 10.1038/s41598-018-24939-2 (PMC5916904; doi:10.1038/s41598-018-24939-2)
Supplement: Supplementary file 1 — Supplementary Figures and Tables [file 41598_2018_24939_MOESM1_ESM.pdf]

Supplementary information

The *Phytophthora cactorum* genome provides insights into the adaptation to host defense compounds and fungicides

Min Yang[1,2†], Shengchang Duan[1,3†], Xinyue Mei[1,2†], Huichuan Huang[1,2,], Wei Chen[1,4], Yixiang Liu[1,2], Cunwu Guo[1,2], Ting Yang[1,2], Wei Wei[1,2], Xili Liu[5], Xiahong He[1,2*], Yang Dong[1,4*], Shusheng Zhu[1,2*]

[1]State Key Laboratory for Conservation and Utilization of Bio-Resources in Yunnan, Yunnan Agricultural University, Kunming, 650201, China

[2]Key Laboratory for Agro-biodiversity and Pest Control of Ministry of Education, Yunnan Agricultural University, Kunming, 650201, China

[3]Nowbio Biotechnology Company, Kunming, 650201, China

[4]Yunnan Research Institute for Local Plateau Agriculture and Industry, Kunming, 650201, China

[5]Department of Plant Pathology, China Agricultural University, Beijing, 100083, China

[*]These authors contributed equally to this work.

[#]Correspondence should be addressed to X.H. (hexiahong@ynau.edu.cn), Y.D. (loyalyang@163.com) or S.Z. (shushengzhu79@126.com).

Supplemental Figure S1 Pathogenicity of *P. cactorum* and other three *Phytophthora* species *P. sojae*, *P. capsici*, and *P. parasitica* to leaf of *P. notoginseng* (a) and their growth profiles on carrot agar medium amended with different concentrations of ginsenosides (b).

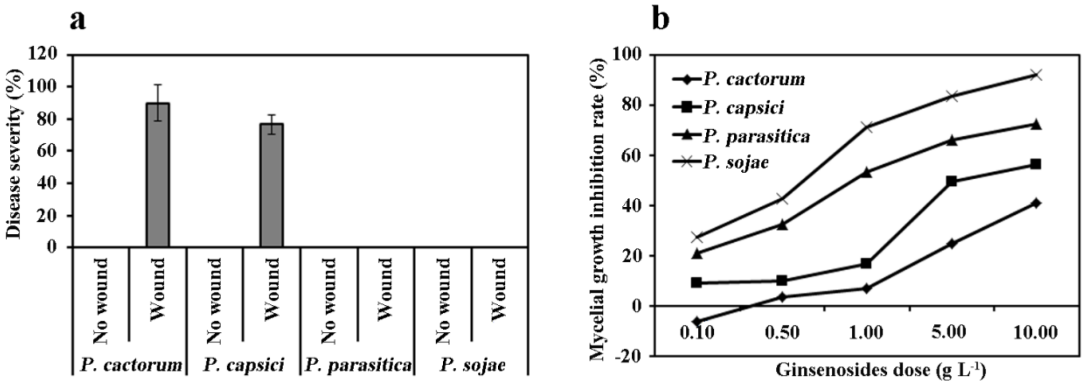

Supplemental Figure S2 Growth profiles of *P. cactorum* and other three *Phytophthora* species *P. sojae*, *P. capsici*, and *P. parasitica* on medium with sole carbon source including glucose, xylan, pectin, cellulose, gum guar or ginsenosides.

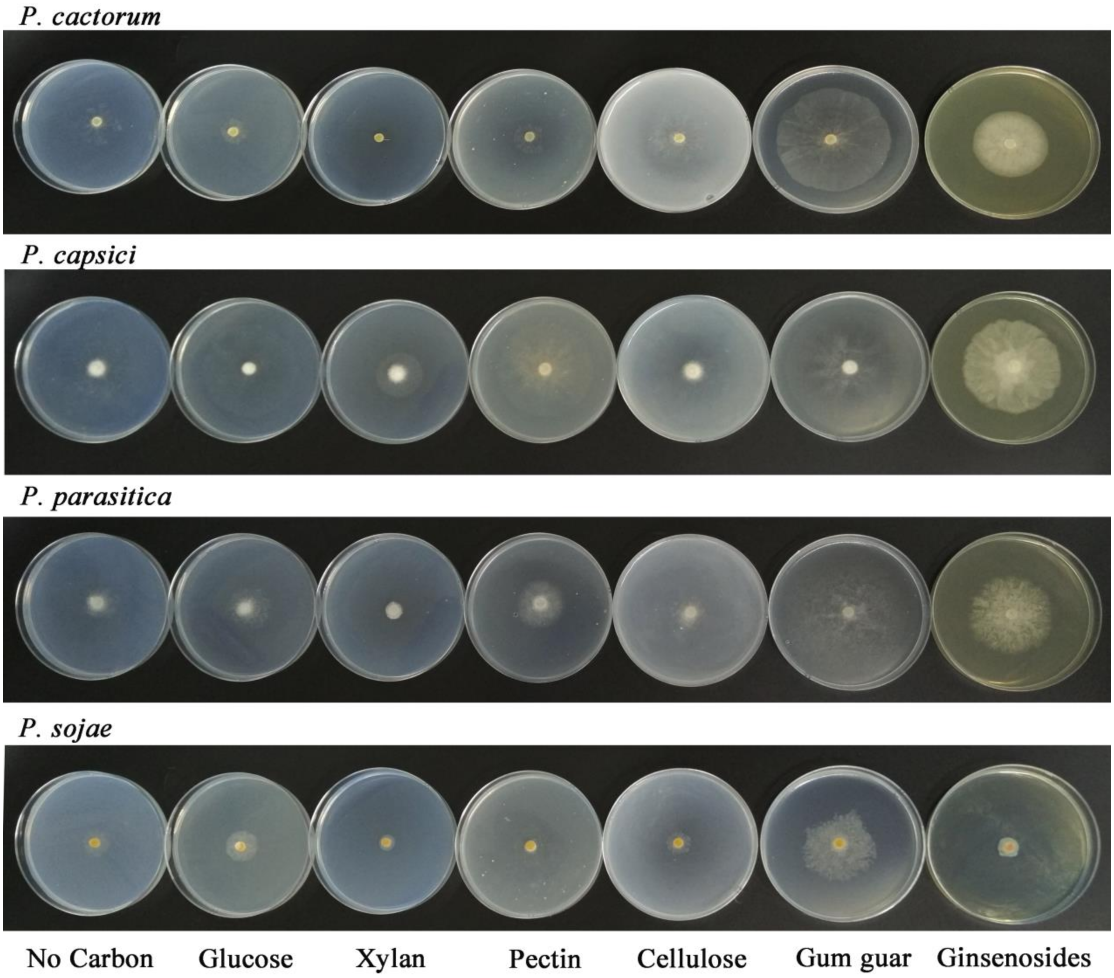

Supplemental Figure S3 Sensitivity of wide host Phytophthora species *P. capsici*, *P. parasitica*, *P. cactorum* and narrow host specie *P. sojae* to commercial fungicides dimethomorph (a), flumorph (b), fluopicolide (c), cymoxanil (d), pyraclostrobine (e), kresoxim-methyl (f), and metalaxyl-M (g).

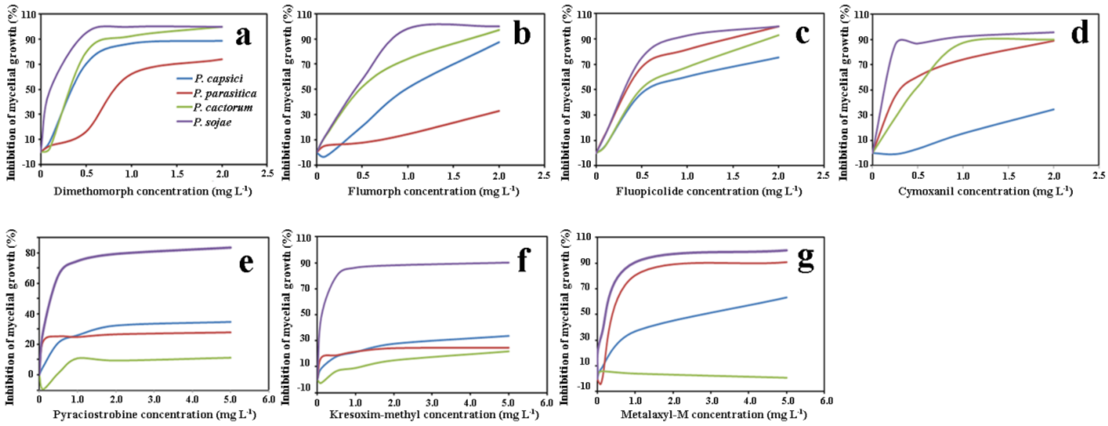

Supplemental Figure S4 The dot plot of the synteny between the genome of four *Phytophthora* genomes. The horizontal coordinate stands for the coordinate of the *P. cactorum* genome, which was sorted according to the scaffold size and laid from left to right. The vertical coordinate is the coordinate of *P. infestans*, *P. sojae* and *P. capsici,* respectively. (a) The dot plot of the synteny between the genome of *P. cactorum* and *P. infestans.* (b) The dot plot of the synteny between the genome of *P. cactorum* and *P. sojae.* (c) The dot plot of the synteny between the genome of *P. cactorum* and *P. capsici.*

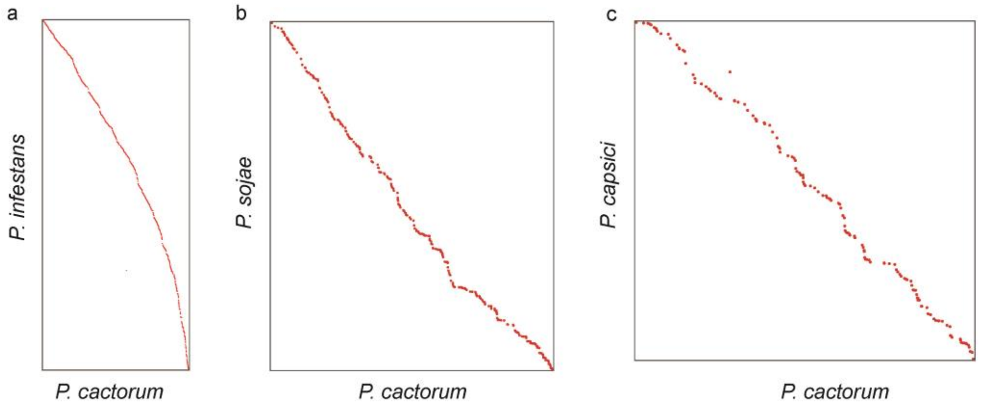

Supplemental Figure S5 The distribution of exon numbers in annotated mRNA. Annotation data of *P. infestans*, *P. sojae*, *P. parasitica* and *P. ramorum* were used in parallel with *P. cactorum* annotation data.

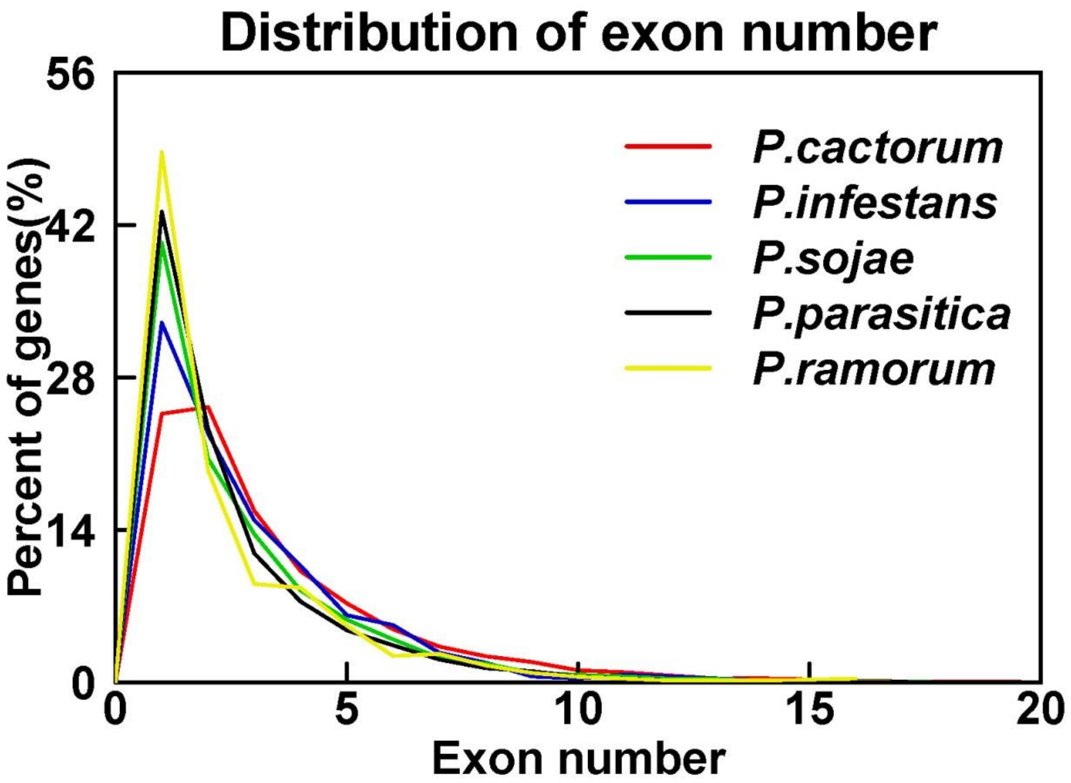

Supplemental Figure S6 Gene characteristic of *P. cactorum.* the distribution of intergenic region lengths in *P. cactorum* genes (a). The distribution of mRNA length (b) and CDS length (c) among *P. infestans*, *P. sojae*, *P. parasitica*, *P. ramorum* and *P. cactorum.* This shows most genes with intergenic regions between 50-bp and 16-kb long.

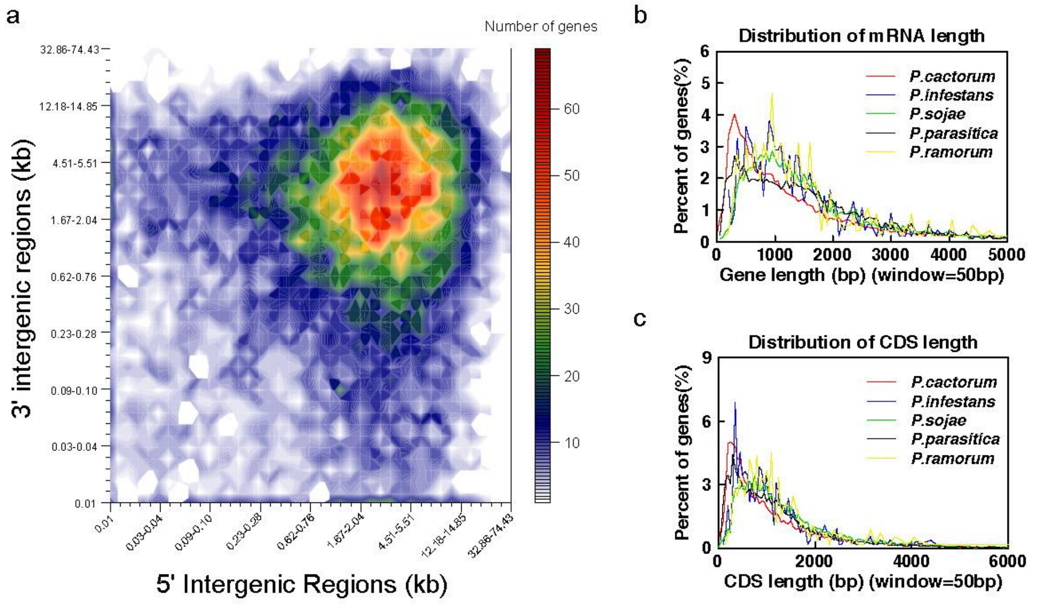

Supplemental Figure S7 Syntenic blocks in *P. cactorum*, genes are shown as red or blue boxes, collinear paralogous gene pairs are connected by chestnut bands. (a) Syntenic blocks between scf0677 and scf2756. (b) Syntenic blocks between scf1586 and scf4776.

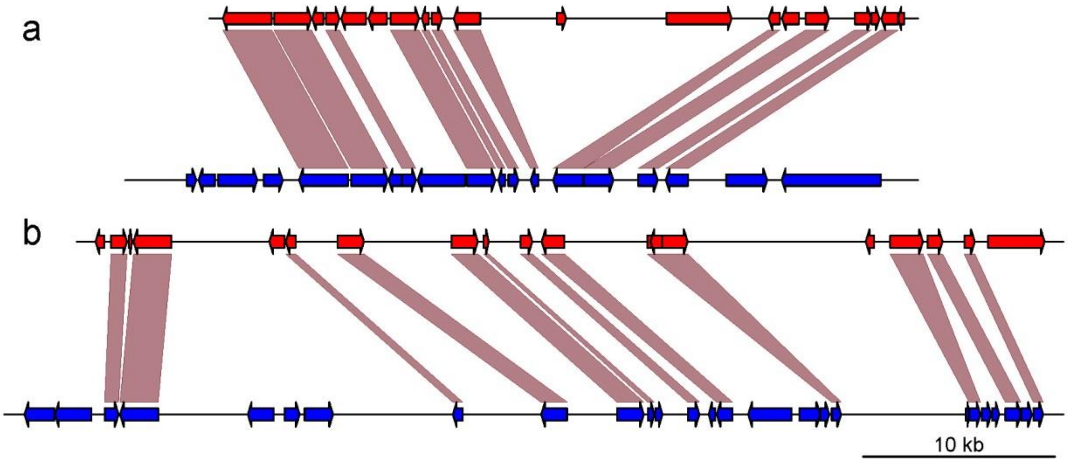

Supplemental Figure S8 Summary for the differentially expressed genes in four *Phytophthora* species after exposure to ginsenosides for 24 h.

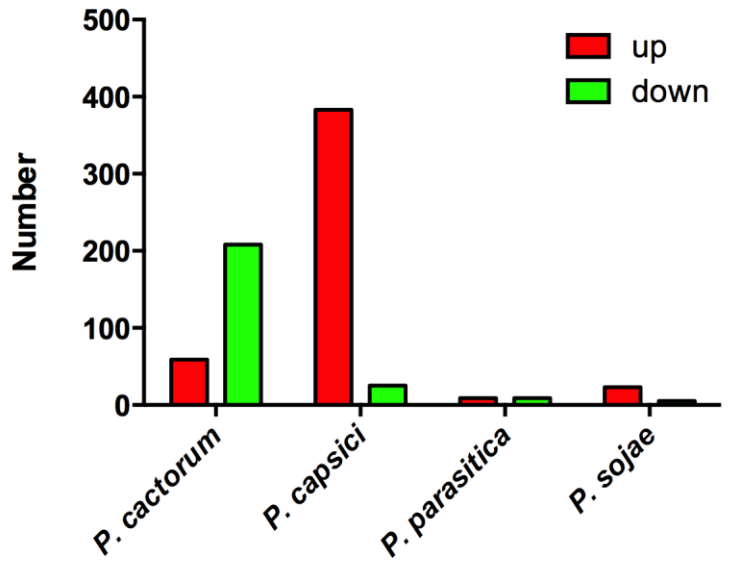

Supplemental Figure S9 Summary for the differentially expressed genes in time-course treatments of *P. cactorum*.

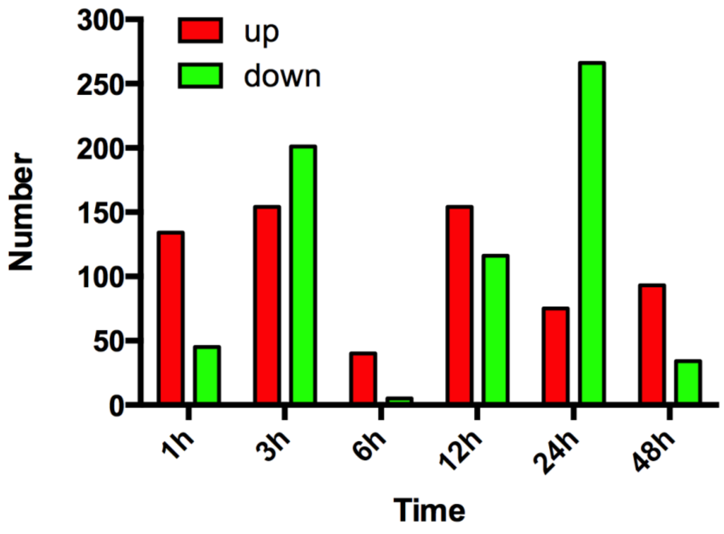

Supplemental Figure S10 GO classification analysis for the differentially expressed genes of *P. cactorum* after exposure to ginsenosides.

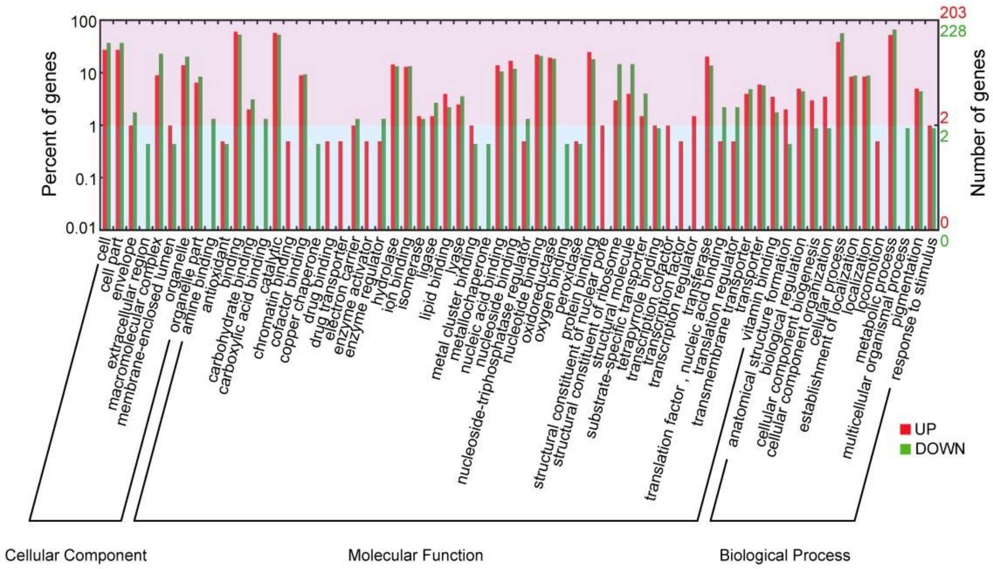

Supplemental Table S1 Summarized benchmarking in BUSCO notation for assembled genome based on Protists dataset*

C:79.1%[S:72.1%,D:7.0%],F:4.2%,M:16.7%,n:215	
Complete BUSCOs (C)	170
Complete and single-copy BUSCOs (S)	155
Complete and duplicated BUSCOs (D)	15
Fragmented BUSCOs (F)	9
Missing BUSCOs (M)	36
Total BUSCO groups searched	215

*Creation date: 2016-11-15, number of species: 33, number of BUSCOs: 215

Supplemental Table S2 Statistics of repeats in *P. cactorum* genome.

Type	Repeat Size (bp)	percent of genome (%)
Trf	7,649,655	6.294664
Repeatmasker	9,815,397	8.076786
Proteinmask	22,422,397	18.450696
De novo	54,245,493	44.636937
Total	56,743,788	46.692706

Supplemental Table S3 TEs content in the assembled *P. cactorum* genome

Type	Repbase TEs		TE proteins		De novo		Combined TEs	
	Length (bp)	% in genome	Length (bp)	% in genome	Length (bp)	% in genome	Length (bp)	% in genome
**DNA**	4,220,164	3.47	6,193,215	5.10	13,847,341	11.39	15,308,382	12.60
**LINE**	68,453	0.06	2,099,343	1.73	3,270,237	2.69	3,671,538	3.02
**SINE**	123,125	0.10	0	0.00	3,326,938	2.74	3,355,116	2.76
**LTR**	5,415,887	4.46	14,165,154	11.66	23,363,796	19.23	24,615,829	20.26
**Other**	0	0.00	0	0.00	0	0.00	0	0.00
**Unknown**	0	0.00	0	0.00	10667003	8.78	10,667,003	8.78
**Total**	9,815,397	8.08	22,422,397	18.45	53,264,049	43.83	55,060,574	45.31

Supplemental Table S7 The predicted miRNA target genes in *P. cactorum*

miRNA_Acc	Target_Acc.	Target start	Target end	miRNA aligned fragment	Target aligned fragment	Inhibition
1	Phyca_scf5131_25397	3396	3416	GUACCAAGCGAACCAGCGCUG	CGGCGCUGGAUCGUUUGGUGC	Cleavage
1	Phyca_scf0457_9654	1748	1768	GUACCAAGCGAACCAGCGCUG	CAAUGCUGCUUUGCUUGGUAC	Cleavage
2	Phyca_scf0667_10695	5739	5759	GCGUUGGUACGCUUGGUAGGC	GCAUACCAAGCGUUCCAAUGC	Cleavage
2	Phyca_scf0465_21450	10	29	GCGUUGGUACGCUUGGUAGG	CCUACCUCGCCUACCAACGC	Translation

Supplemental Table S8 Overview of the gene family clustering in eight sequenced *Phytophthora* species.

Species	Genes number	Genes in families	Unclustered genes	Family number	Unique families	Average genes per family
P. infestans	17,787	15,629	2,158	10,828	232	1
P. kernoviae	10,650	8,302	2,348	7,056	54	1
P. lateralis	11,635	9,360	2,275	7,653	75	1
P. parasitica	28,117	22,509	5,608	12,613	538	2
P. ramorum	15,605	14,583	1,022	9,600	179	2
P. sojae	18,969	16,796	2,173	10,240	338	2
P. cactorum	27,981	19,783	8,198	11,674	893	2
P. capsici	19,805	14,794	5,011	9,841	580	2
