# Supplementary material for: The Phytophthora cactorum genome provides insights into the adaptation to host defense compounds and fungicides
Source: Sci Rep. 2018 Apr 25;8:6534. doi: 10.1038/s41598-018-24939-2 (PMC5916904; doi:10.1038/s41598-018-24939-2)
Supplement: Supplementary file 2 — Supplementary Dataset Information [file 41598_2018_24939_MOESM2_ESM.docx]

***Supplementary Dataset information***

**The** ***Phytophthora cactorum* genome provides insights into the adaptation to host defense compounds and fungicides**

Min Yang^1,2†^, Shengchang Duan^1,3†^, Xinyue Mei^1,2†^, Huichuan Huang^1,2,^, Wei Chen^1,4^, Yixiang Liu^1,2^, Cunwu Guo^1,2^, Ting Yang^1,2^, Wei Wei^1,2^, Xili Liu^5^, Xiahong He^1,2*^, Yang Dong^1,4*^, Shusheng Zhu^1,2*^

^1^State Key Laboratory for Conservation and Utilization of Bio-Resources in Yunnan, Yunnan Agricultural University, Kunming, 650201, China

^2^Key Laboratory for Agro-biodiversity and Pest Control of Ministry of Education, Yunnan Agricultural University, Kunming, 650201, China

^3^Nowbio Biotechnology Company, Kunming, 650201, China

^4^Yunnan Research Institute for Local Plateau Agriculture and Industry, Kunming, 650201, China

^5^Department of Plant Pathology, China Agricultural University, Beijing, 100083, China

^*^These authors contributed equally to this work.

^#^Correspondence should be addressed to X.H. (hexiahong@ynau.edu.cn), Y.D. (loyalyang@163.com) or S.Z. ([shushengzhu79@126.com](mailto:shushengzhu79@126.com)).

**Supplementary Table S4** FPKM values of genes.

**Supplementary Table S5** Overview of genes functional annotation against NR, Swiss-Prot, KEGG and InterProScan database.

**Supplementary Table S6** Annotated ncRNA in the genome of *P. cactorum, P. infestans, P. ramorum* and *P. sojae.*

**Supplementary Table S9** GO enriched results of unique paralogous genes in *P. cactorum* compared to eight other sequenced *Phytophthora* species.

**Supplementary Table S10** GO enriched results of genes undergone expansion in *P. cactorum* compared to 15 other species in Figure 2.

**Supplementary Table S11** GO enriched results of genes undergone contraction in *P. cactorum* compared to 15 other species in Figure 2.

**Supplementary Table S12** The positively selected genes of *P. cactorum* against *P. infestans, P. lateralis, P. capsici, P. ramorum, P. kernoviae, P.parasitica* and *P. sojae.*

**Supplementary Table S13** GO enriched results of positive selectived genes of *P. cactorum* compared to 7 *Phytophthora* species.

**Supplementary Table S14** The differentially expressed genes in *P. cactorum* angaist ginsenoside for 24h.

**Supplementary Table S15** The differentially expressed genes in *P. capsici* angaist ginsenoside for 24h.

**Supplementary Table S16** The differentially expressed genes in *P. parasitica* angaist ginsenoside for 24h.

**Supplementary Table S17** The differentially expressed genes in *P. sojae* angaist ginsenoside for 24h.

**Supplementary Table S18** The differentially up-expressed genes in *P. cactorum* angaist time-course ginsenoside treatment.

**Supplementary Table S19** The differentially down- expressed genes in *P. cactorum* angaist time-course ginsenoside treatment.

**Supplementary Table S20** The identified transcription factor in *P. cactorum*, *P. parasitica*, *P. infestans*, *P. kernoviae*, *P. ramorum*, *P. sojae* and *P. capsici*.

**Supplementary Table S21** The identified protein kinases in *P. cactorum,* *P. parasitica, P. infestans*, *P. kernoviae*, *P. ramorum*, *P. sojae* and *P. capsici.*
